# Effect of ribose-glycated BSA on histone demethylation

**DOI:** 10.3389/fgene.2022.957937

**Published:** 2022-10-05

**Authors:** Mengqi Xi, Lingyun Zhang, Yan Wei, Ting Li, Meihua Qu, Qian Hua, Rongqiao He, Ying Liu

**Affiliations:** ^1^ School of Life Sciences, Beijing University of Chinese Medicine, Beijing, China; ^2^ Institute of Biophysics, Chinese Academy of Sciences, Beijing, China; ^3^ Bayannur Hospital, Bayannur, China; ^4^ Second People’s Hospital of Weifang, Weifang, Shandong, China

**Keywords:** ribose, histone methylation, LSD1, glucose, glycation, advanced glycation end-products

## Abstract

A reducing sugar reacts with the protein, resulting in advanced glycation end-products (AGEs), which have been implicated in diabetes-related complications. Recently, it has been found that both type 1 and type 2 diabetic patients suffer from not only glucose but also ribose dysmetabolism. Here, we compared the effects of ribose and glucose glycation on epigenetics, such as histone methylation and demethylation. To prepare ribose-glycated (riboglycated) proteins, we incubated 150 μM bovine serum albumin (BSA) with 1 M ribose at different time periods, and we evaluated the samples by ELISAs, Western blot analysis, and cellular experiments. Riboglycated BSA, which was incubated with ribose for approximately 7 days, showed the strongest cytotoxicity, leading to a significant decrease in the viability of SH-SY5Y cells cultured for 24 h (IC_50_ = 1.5 μM). A global demethylation of histone 3 (H3K4) was observed in SH-SY5Y cells accompanied with significant increases in lysine-specific demethylase-1 (LSD1) and plant homeodomain finger protein 8 (PHF8) after treatment with riboglycated BSA (1.5 μM), but demethylation did not occur after treatment with glucose-glycated (glucoglycated) proteins or the ribose, glucose, BSA, and Tris–HCl controls. Moreover, a significant demethylation of H3K4, H3K4me3, and H3K4me2, but not H3K4me1, occurred in the presence of riboglycated proteins. A significant increase of formaldehyde was also detected in the medium of SH-SY5Y cells cultured with riboglycated BSA, further indicating the occurrence of histone demethylation. The present study provides a new insight into understanding an epigenetic mechanism of diabetes mellitus (DM) related to ribose metabolic disorders.

## Graphical Abstract

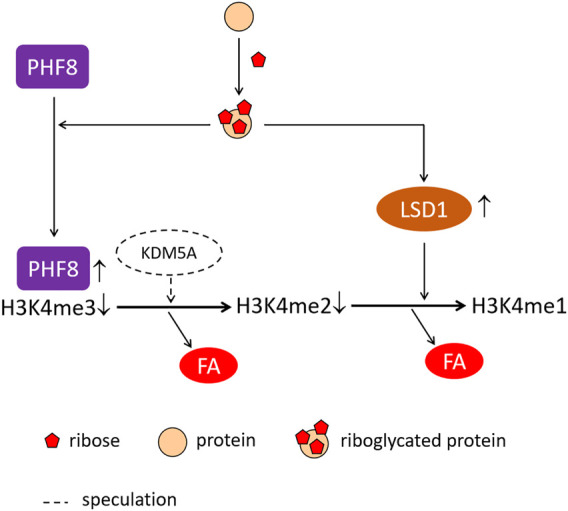

## Introduction

In 1815, the French chemist, M.E. Chevreul (1786–1889), discovered that the sweetness in the urine of diabetics comes from grape sugar or D-glucose (Fournier, 2011). Diabetes mellitus (DM) is considered a group of metabolic diseases characterized by hyperglycemia (high concentration of blood D-glucose), resulting from defects in insulin secretion, insulin action, or both. Ribose has been neglected until 2013 when Su et al. (2013)first detected high levels of ribose in the urine of diabetics. After that, high blood ribose levels in both type 1 (Yu et al., 2019) and type 2 (Chen et al., 2019) diabetes have also been observed through clinical investigations. Mou et al. (2022) showed that ribose selectively glycates some lysine residues in bovine serum albumin (BSA) in comparison to glucose. Animal studies have also demonstrated high blood ribose levels in Zucker diabetic fatty rats (Chen et al., 2017), streptozotocin (STZ)-induced type 1 diabetes (T1D) rats (Yu et al., 2019), STZ-induced type 2 diabetes (STZ-T2D) rats (unpublished data), and Goto–Kakizaki (GK) rats (Cao et al., 2022) compared to controls. These data suggest that diabetic patients suffer from not only glucose dysmetabolism but also ribose dysmetabolism.

The reactions that lead to the formation of advanced glycation end-products (AGEs) have been known for over a hundred years since Maillard’s description in 1912 (Maillard, 1912). A chronic hyperglycemic state promotes the formation of AGEs from non-enzymatic glycation of proteins, lipids, and nucleic acids in patients with DM, aggravating multiple diabetic complications (Fournier, 2011; Jud and Sourij., 2019; Khalid et al., 2022; Twarda-Clapa et al., 2022). In recent years, the relationship between glycation and epigenetics has received increasing attention (Perrone et al., 2020). Elucidation of the pathways and mechanisms of diabetic complications, resulting from hyperglycemia through epigenetic events, will aid in the prevention and treatment of diabetes.

Post-translational modifications (PTMs) of histone are powerful epigenetic mechanisms. Histone methylation can cause either relaxation or condensation of chromatins, thereby causing the activation or repression of transcription factors (Yang et al., 2020; Yamunadevi et al., 2021). The methylation process is regulated by a balance between histone-methylating and -demethylating enzymes (Chang et al., 2021). Histone demethylases are classified into two families according to their catalytic mechanisms, namely, lysine-specific demethylases (LSDs) and Jumonji C-terminal-containing histone demethylases (JmjC demethylases) (Kim et al., 2022). The removal of methyl groups from methylated H3K4me1/2 by LSD1 (KDM1A) induces transcriptional repression, while that from methylated H3K9 induces transcriptional activation (Forneris et al., 2008). Plant homeodomain (PHD) finger protein 8 (PHF8) binds to H3K4me3-marked nucleosomes and acts as a demethylase, specific for H3K9me2 (Fortschegger et al., 2010; Liu et al., 2021).

Hyperglycemia induces a dynamic co-operativity of histone methylase and demethylase enzymes associated with gene-activating epigenetic marks (Brasacchio et al., 2009). These events may integrate and result in sustained activation of pro-inflammatory pathways, which are involved in the progression of diabetic complications (Sun et al., 2014). Histone methylation of retinal Sod2 has an important role in the development of diabetic retinopathy and the metabolic memory phenomenon associated with its continued progression (Zhong and Kowluru., 2013). H3K4 methylation has been implicated in the dysregulation of critical genes involved in the progression of diabetic nephropathy (DN) (Yang et al., 2022). Li et al. (2021) revealed that ribose-induced nephropathy (mesangial cell injury) results from the receptor of AGE (RAGE)-dependent NF-kB inflammation (Hong et al., 2018; Zhang et al., 2019). Targeting enzymes important for histone methylation may serve as a potential therapy to halt the development of DN and diabetic retinopathy, indicating that changes in epigenetic marks that coexist on the lysine tail during glycemia need additional investigation.

Thus far, glucose glycation (glucoglycation) has been thoroughly investigated (Brasacchio et al., 2009; Ding and Huang., 2014). However, the effects of ribose glycation (riboglycation) on genetics and epigenetics of metabolic disorders need to be clarified, especially in hyperglycemia. Histone demethylation produces formaldehyde (Shi et al., 2004), and we have observed that urine formaldehyde levels are negatively correlated with age-related cognitive abilities and education level in the elderly community (Yu et al., 2014). We have also reported a negative correlation between urine formaldehyde levels and Mini-Mental State Examination (MMSE) scores (Tong et al., 2011) as well as an association of urine formaldehyde levels with DNA de/methylation (Tong et al., 2015). Thus, formaldehyde and its metabolism may be related to de/methylation and age-related metabolic disorders (He, 2019; Li et al., 2021). In the present study, we compared the effects of ribose-glycated (riboglycated) and glucose-glycated (glucoglycated) proteins on histone de/methylation in SH-SY5Y cells. A decrease in the global methylation of H3K4 was observed followed by demethylation of H3K4me3 and H3K4me2 with a significant upregulation of LSD1 and PHF8. At the same time, formaldehyde levels increased in the culture medium. Overall, these findings suggested that riboglycation plays a role in epigenetic diabetes-like disorders.

## Materials and methods

### Antibodies

The following primary antibodies were used: anti-AGEs (1:2000, 6D12, Trans Genic Inc., Japan), H3K4me1 (1:10000, ab176877, Abcam, United States), H3K4me2 (1:10000, ab32356, Abcam, United States), H3K4me3 (1:5000, ab213224, Abcam, United States), histone H3 (1:10000, ab176842, Abcam, United States), LSD1 (1:2500, 2184, Cell Signaling Technology, United States), PHF8 (1:5000, ab280887, Abcam, United States), Bax (1:5000, 50599-2-Ig, Proteintech, China), Bcl-2 (1:4000, A0208, Abclonal, China), and β-actin (1:2000, K101527P, Solarbio, China).

### *In vitro* preparation of riboglycated BSA

BSA (Sigma, United States) was dissolved in 20 mM Tris–HCl buffer (pH 7.4) to a final concentration of 150 μM, and it was mixed with different final concentrations (0.1, 0.5, and 1 M) of ribose (Lab Lead, China) or glucose (Ameresco, United States). As controls, 150 μM BSA, 1 M ribose, and 1 M glucose were separately dissolved in Tris–HCl buffer. All solutions were filtered using 0.22-μm membranes and then incubated at 37°C for 7 days without light. Aliquots were collected for measurements at different time periods from Day 1 to Day 7. The incubated products were stored at −20°C, and repeated freeze–thaw was avoided.

### Western blot analysis

Protein quantification was performed using a BCA protein assay kit (Thermo Fisher, United States). The protein samples with an adjusted concentration were mixed with 5×SDS loading buffer and boiled for 10 min at 100°C. Proteins (3–30 μg) (Butler et al., 2019; Abcam, 2022) were separated by 12% SDS-PAGE gels and then transferred onto PVDF membranes (Millipore, United States) at 4°C. The membranes were blocked with 5% skim milk in TBS containing 0.1% Tween-20 (TBST, pH 7.4) at room temperature for 1 h followed by incubation with primary antibodies for 2 h under the same conditions. The membranes were then washed three times with TBST and incubated with goat anti-mouse or anti-rabbit horseradish peroxidase (HRP) (1:5000, KPL, United States) at room temperature for 2 h. After three washes with TBST, the immunoreactive bands were visualized by enhanced chemiluminescence (e-Blot Touch Imager, China), and the results were quantified using ImageJ software (http://ImageJ.nih.gov).

### Advanced glycation end-product quantitative detection by enzyme-linked immunosorbent assay

The formation of AGEs was measured using an advanced glycation end-product (AGE) enzyme-linked immunosorbent assay (ELISA) kit (FineTest, China). In brief, the plate was washed twice with washing buffer before detection, and 50 μl of the sample or standard was added to each well followed immediately by the addition of 50 μl of biotin-labeled antibody. The plate was gently mixed and then incubated at 37°C for 45 min. After washing three times, 100 μl of SABC working solution was added to each well and incubated at 37°C for 30 min. After five washes, the plate was incubated with 90 μl of TMB substrate solution in the dark at 37°C for 15 min, and 50 μl of stop solution was then added. The absorbance was immediately measured at 450 nm using a microplate reader (Molecular Devices, United States).

### Cell culture and treatment with riboglycated protein

The human SH-SY5Y neuroblastoma cell line was obtained from the Institute of Basic Medical Sciences of the Chinese Academy of Medical Sciences (Beijing, China). The cells were cultured in Dulbecco’s modified Eagle’s medium (DMEM) (Gibco, United States) supplemented with 10% fetal bovine serum (FBS) (ExCell Bio, Australia), 0.1 U L^−1^ penicillin, and 0.1 g L^−1^ streptomycin at 37°C in a humidified chamber with 5% CO_2_. The cells were grown to 80–90% confluence in 100-mm-diameter dishes and subcultured every 2 days. For all experiments, the medium was replaced with a serum-free medium before treatment with incubation products.

BSA incubated with 1 M ribose (or 1 M glucose) for 7 days was diluted with DMEM and used for the treatment of cells. As controls, 150 µM BSA alone, 1 M ribose alone, or 1 M glucose alone was employed in the cell experiments, and 20 mM Tris–HCl buffer was used as a blank control.

### Cell viability detection

Cell viability was detected using a cell counting kit-8 assay (CCK-8) (Dojindo, Japan). The SH-SY5Y cells were plated in 96-well plates at a concentration of 10^4^ cells per well. After 24 h, different concentrations of riboglycated BSA (0, 0.6, 1.2, 1.5, 1.8, 2.4, and 3 μM) were added to SH-SY5Y cells followed by measurements of cell viability after 24 h. In another experiment, 1.5 μM riboglycated BSA was added to cells 24 h after plating for different time intervals (0, 6, 12, 24, 36, 48, 60, and 72 h) followed by measurements of cell viability. After treatment, the CCK-8 reagent was added, and the plates were incubated at 37°C for 1.5 h. Under the same conditions, the cells were treated with glucoglycated BSA in addition to the ribose, glucose, BSA, and Tris–HCl controls. The absorbance was recorded at 450 nm using a SpectraMax Paradigm microplate reader (Molecular Devices, United States).

### Cell apoptosis analysis by flow cytometry

The SH-SY5Y cells were plated in six-well plates at a concentration of 1.5 × 10^5^ cells per well and cultured with 1.5 μM riboglycated BSA, 1.5 μM glucoglycated BSA, 1.5 μM BSA, 10 mM ribose, or 10 mM glucose alone for 24 h. The cells treated with Tris–HCl buffer were used as a control. Cell apoptosis was detected using an Annexin V-FITC/PI apoptosis detection kit (Beyotime, China). In brief, the cells were digested by trypsin without EDTA and washed twice with PBS. The cells (10^4^) suspended with Annexin V-FITC binding solution were transferred to flow cytometry tubes and gently mixed with 4 μl of Annexin V-FITC and 10 μl of propidium iodide (PI) staining solution. The cells were then incubated at room temperature (20–25°C) for 10–20 min in the dark. Apoptotic cells were then determined by flow cytometry (BD FACSCalibur, United States), and the data were analyzed using FlowJo 7.6.1 software (TreeStar Inc., OR).

### Histone extraction and global histone H3-K4 methylation detection

The experimental conditions were the same as those described previously for the cell apoptosis experiments. A global histone H3-K4 methylation assay kit (Epigentek, United States) was used to extract histones and quantify global histone H3-K4 methylation of SH-SY5Y cells according to the manufacturer’s instructions.

### Lysine-specific demethylase-1 quantitative detection by enzyme-linked immunosorbent assay

The experimental conditions were the same as those described previously for the cell apoptosis experiments. Lysine-specific demethylase-1 was assayed using a Human KDM1A (Lysine-specific histone demethylase 1A) ELISA Kit (FineTest, China) according to the manufacturer’s instructions.

### Measurements of formaldehyde by high-performance liquid chromatography with UV detection

Formaldehyde in the medium of SH-SY5Y cells, incubated with riboglycated BSA, was quantified by high-performance liquid chromatography with UV detection (UV-HPLC). The samples of the culture medium were centrifuged (13,000 rpm, 4°C, 20 min), and the supernatants (0.4 ml) were transferred to 1.5-ml Eppendorf tubes and mixed with 0.1 ml of 10% trichloroacetic acid, 0.4 ml of acetonitrile, and 0.1 ml of 2,4-dinitrophenylhydrazine (DNPH, 0.1 g L^−1^). The mixture was centrifuged (13,000 rpm, 4°C, 10 min) and incubated at 60°C for 30 min followed by cryogenic centrifugation to stop the reaction. The samples were filtered through 0.22-μm membranes and detected using an HPLC system (LC-20A, Japan) as described previously (Su et al., 2011).

### Data analysis

All data are presented as the mean ± SEM, and all statistical analyses were performed using GraphPad Prism 7.0 software (La Jolla, United States). Statistical significance was analyzed by one-way or two-way analysis of variance (ANOVA) followed by Tukey’s test for multiple comparisons. The statistical significance was set at *p* < 0.05.

## Results

### Riboglycation occurs more rapidly than glucoglycation

**FIGURE 1:**
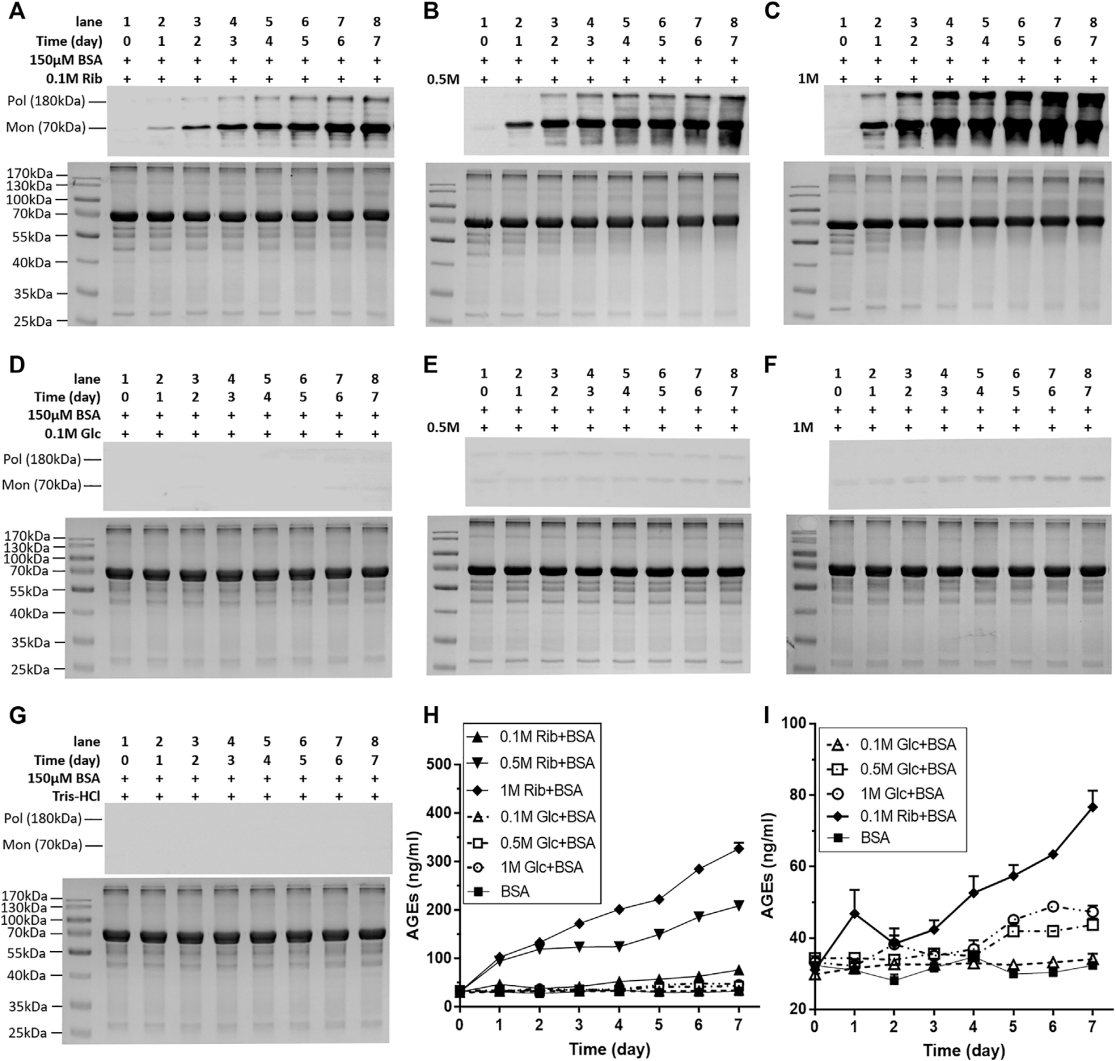
Change in AGEs of BSA in the presence of different concentrations of ribose or glucose according to Western blot analysis and ELISAs. BSA (final concentration, 150 μM) was incubated with different concentrations of ribose (0.1, 0.5, and 1 M) or glucose (0.1, 0.5, and 1 M) in Tris–HCl buffer (20 mM, pH 7.4) at 37°C for 7 days. Aliquots were taken at different time periods for measurements. AGEs were detected by Western blot analysis **(A–G)** and ELISAs **(H,I)**. BSA alone was used as the control. **(I)** Magnification of the y-axis in **(H)**. Data shown in **(H,I)** are presented as the mean ± SEM (*n* = 3). Abbreviations: Rib, ribose; Glc, glucose; Mon, monomer; Pol, polymer.

To investigate whether riboglycated protein affects histone methylation, we utilized BSA because it is commonly used as a model in glycation studies. Before determining whether changes in histone methylation induced by riboglycated proteins are different from those induced by glucoglycated proteins, we compared the BSA-glycating and AGE-producing abilities of ribose and glucose at different concentrations (Figure 1). Western blot analysis evidently exhibited that compared with those of glucose, different concentrations of ribose (0.1, 0.5, and 1 M) incubated with BSA could significantly produce many AGEs (Figures 1A–C). AGEs were visualized during the riboglycation of BSA on Day 1 and Day 2 when 0.5 and 0.1 M ribose was used, respectively. However, glucoglycation did not produce AGEs at Day 1 or Day 2, except for slight protein bands with high glucose concentration (1 M) under the same experimental conditions (Figures 1D–F). No AGEs were observed during the incubation of BSA with Tris–HCl as the control (Figure 1G). The riboglycated BSA was prone to aggregation during incubation, resulting in polymers with a molecular mass greater than 180 kDa. A BSA monomer (approximately 70 kDa) was also observed in the SDS-PAGE and Western blot analyses. The ELISA results demonstrated that significantly more AGEs resulted from riboglycation than glucoglycation at different concentrations for different time periods (Figure 1H).

**FIGURE 2:**
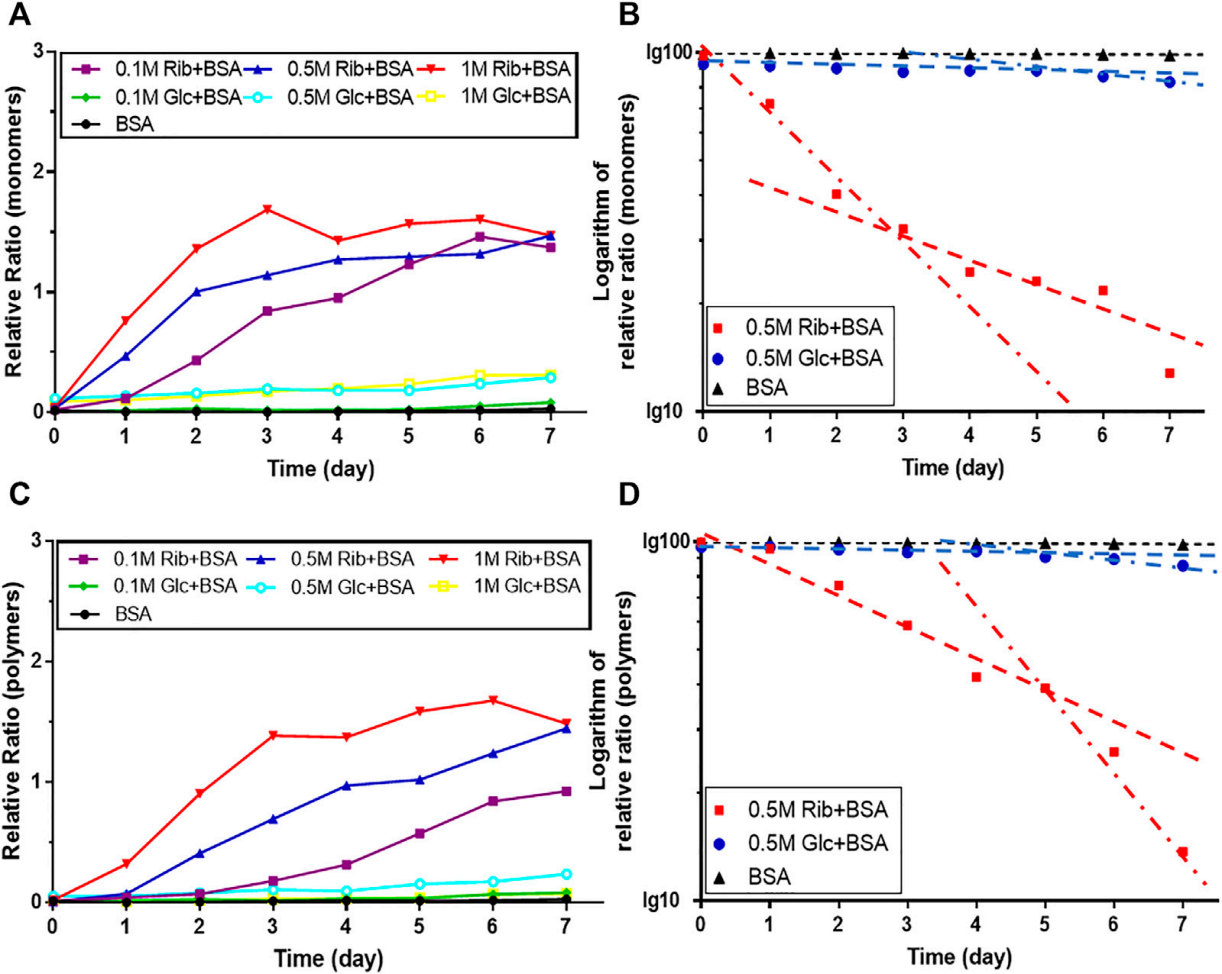
Kinetic changes in AGEs after the incubation of BSA with ribose. The same data (BSA incubated with 0.5 M ribose or 0.5 M glucose) from Figure 1 were analyzed by Tsou’s method. Blotting densities of monomers are shown in **(A)**, and data were plotted in semi-logarithmic coordinates **(B)**. Blotting densities of polymers are shown in **(C)**, and data were plotted in semi-logarithmic coordinates **(D)**.

In the kinetic study of riboglycation, we scanned the densities of protein blots at each time point and observed increases in AGEs in monomers (Figures 2A,B) and polymers (Figures 2C,D) over the incubation time. However, we did not observe a greatly increasing rate of AGEs when BSA was incubated with different concentrations of glucose (0.1, 0.5, and 1 M). We utilized Tsou’s method (Tsou, 1962) to calculate and estimate the rate of BSA glycation of ribose. As shown in Figure 2B, the riboglycation of BSA monomers underwent a biphasic procedure (fast and slow phase), and the riboglycation of BSA polymers also underwent a biphasic procedure (slow and fast phase) (Figure 2D) with a relaxation time of approximately 1 day. The first-order rate constant of riboglycation in monomers in the fast phase for monomers (6.71 × 10^−6^ s^−1^) was similar to that for polymers (3.13 × 10^−6^ s^−1^), indicating that AGEs resulting from riboglycation first appear in BSA monomers and then aggregate into polymers. The comparison of the kinetic formation of AGEs showed that the first-order rate constants of riboglycation in slow and fast phases were approximately 15.5- and 7.8-fold faster than those for glucoglycation in monomers, and that they were 17.2- and 6.3-fold faster than those for glucoglycation in polymers, respectively (Table 1). Thus, these findings indicated that ribose actively participates in protein glycation and produces AGEs more rapidly than glucose at different concentrations (0.1, 0.5, and 1 M).

**TABLE 1:** First-order rate constants of BSA riboglycation and glucoglycation as calculated by Tsou’s method (Tsou, 1962).

Rate constant (× 10^−6^ s^−1^)	Polymer (180 kDa)		Monomer (70 kDa)	
	Slow	Fast	Slow	Fast
K_0.1R_	0.205	2.831	1.636	6.876
K_0.5R_	1.593	3.131	2.502	6.711
K_R_	2.325	11.015	4.190	17.682
K_0.1G_	0.079	—	0.076	—
K_0.5G_	0.093	0.495	0.161	0.863
K_G_	0.052	—	0.158	0.603
K_BSA_	0.016	—	0.013	—
K_0.1R_/K_0.1G_	2.609	—	21.612	—
K_0.5R_/K_0.5G_	17.172	6.324	15.536	7.780
K_R_/K_G_	44.491	—	26.513	29.306

Analysis was based on the data in Figure 1. K_0.1R_, K_0.5R_, and K_R_ indicate the constants of 0.1, 0.5, and 1 M ribose incubated with BSA, respectively; K_0.1G_, K_0.5G_, and K_G_ indicate the constants of 0.1, 0.5, and 1 M glucose incubated with BSA, respectively; K_BSA_ indicates the constant of BSA alone; K_0.1R_/K_0.1G_ indicates the ratio of K_0.1R_ to K_0.1G_; K_0.5R_/K_0.5G_ indicates the ratio of K_0.5R_ to K_0.5G_; and K_R_/K_G_ indicates the ratio of K_R_ to K_G_.

### Riboglycated protein is more cytotoxic than glucoglycated protein

**FIGURE 3:**
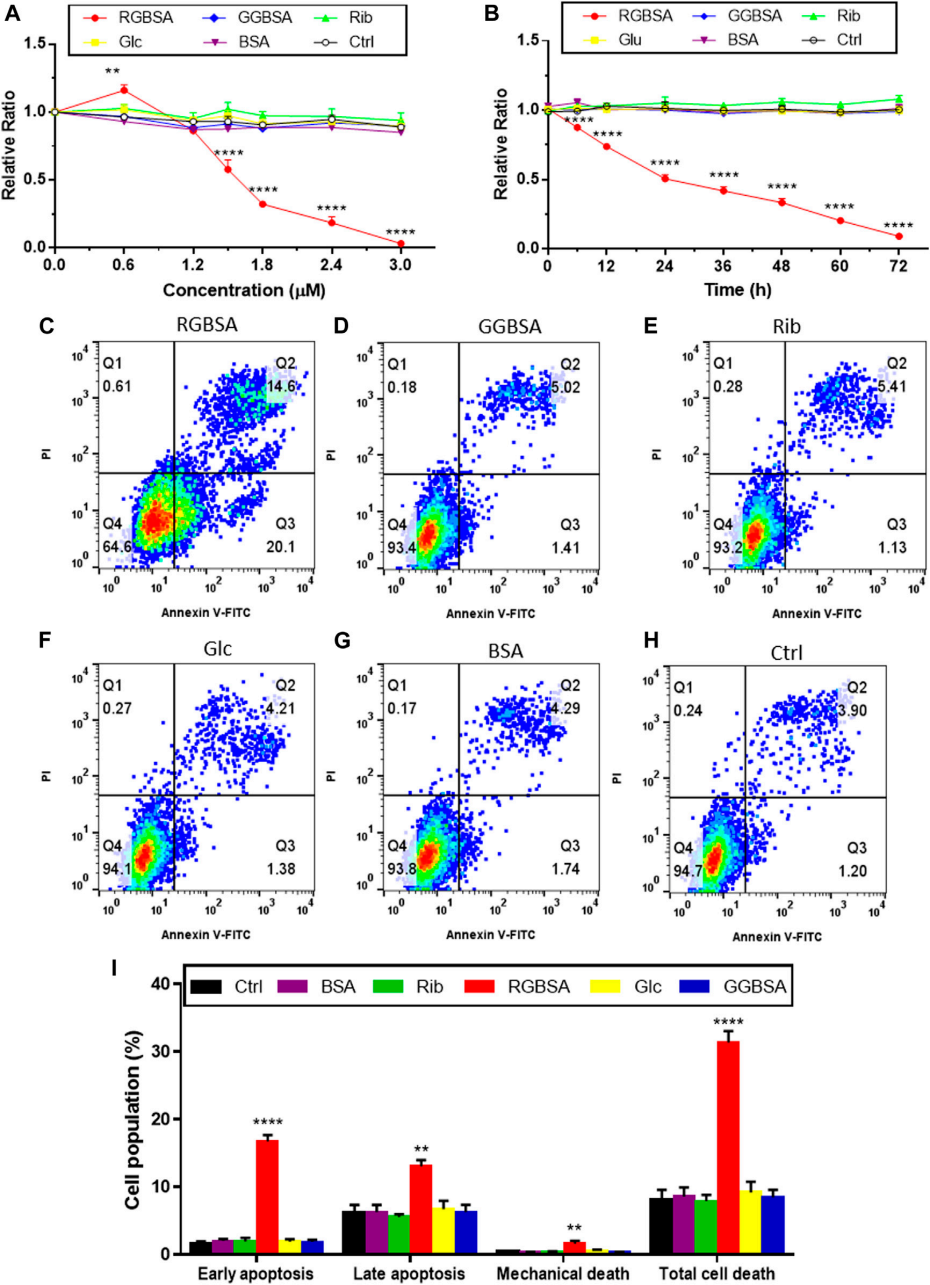
Riboglycated BSA induces SH-SY5Y cell apoptosis. Experimental conditions were the same as in Figure 1, except for SH-SY5Y cells that were incubated with different concentrations of riboglycated BSA (0, 0.6, 1.2, 1.5, 1.8, 2.4, and 3 μM) followed by the measurement of cell viability after 24 h **(A)** or SH-SY5Y cells that were incubated with 1.5 μM riboglycated BSA for different time periods (0, 6, 12, 24, 36, 48, 60, and 72 h) followed by the measurement of cell viability **(B)**. Data were analyzed by two-way ANOVA and are presented as the mean ± SEM (*n* = 3). **p < 0.01 and ****p < 0.0001 compared to the control group. Flow cytometry was used to assess apoptosis of SH-SY5Y cells cultured for 24 h in the presence of 1.5 μM RGBSA **(C)**, GGBSA **(D)**, ribose **(E)**, glucose **(F)**, BSA **(G),** and Tris–HCl buffer **(H)**. Percentages of apoptosis were determined by Annexin V/FITC-PI staining **(I)**. Data were analyzed by one-way ANOVA and are presented as the mean ± SEM (*n* = 3). **p < 0.01 and ****p < 0.0001 compared to the control group. Abbreviations: RGBSA, riboglycated BSA; GGBSA, glucoglycated BSA.

Because riboglycation is faster than glucoglycation, we next investigated whether ribose-mediated AGEs are more toxic than glucose-mediated AGEs by evaluating SH-SY5Y cell viability after culture in the presence of riboglycated or glucoglycated BSA. Here, riboglycated BSA was the product resulting from the incubation of BSA with ribose for 7 days unless otherwise stated. As shown in Figure 3A, the viability of SH-SY5Y cells was significantly decreased as the concentration of riboglycated BSA increased, but the viability was not affected by glucoglycated BSA, ribose alone, glucose alone, or BSA alone. The IC_50_ of the inhibitory effect of riboglycated BSA on cell viability was approximately 1.5 μM. Thus, we cultured SH-SY5Y cells with riboglycated BSA at a final concentration of 1.5 μM for different time periods, and we found a significant decrease in cell viability in the presence of riboglycated BSA over time (Figure 3B). Moreover, glucoglycated BSA and the ribose, glucose, BSA, and Tris–HCl buffer controls did not significantly affect cell viability. Thus, these data indicated that riboglycated BSA is more cytotoxic than glucoglycated BSA.

Because we demonstrated that riboglycated BSA was cytotoxic, we used flow cytometry to assess the characteristics of SH-SY5Y cell death in the presence of riboglycated or glucoglycated BSA. As shown in Figure 3C, riboglycated BSA caused a significant increase in early apoptotic (Q3) SH-SY5Y cells after 24 h. In addition, there were more necrotic and late apoptotic (Q2) SH-SY5Y cells treated with riboglycated BSA compared to those treated with glucoglycated BSA (Figure 3D) and the controls (Figures 3E–H).

**FIGURE 4:**
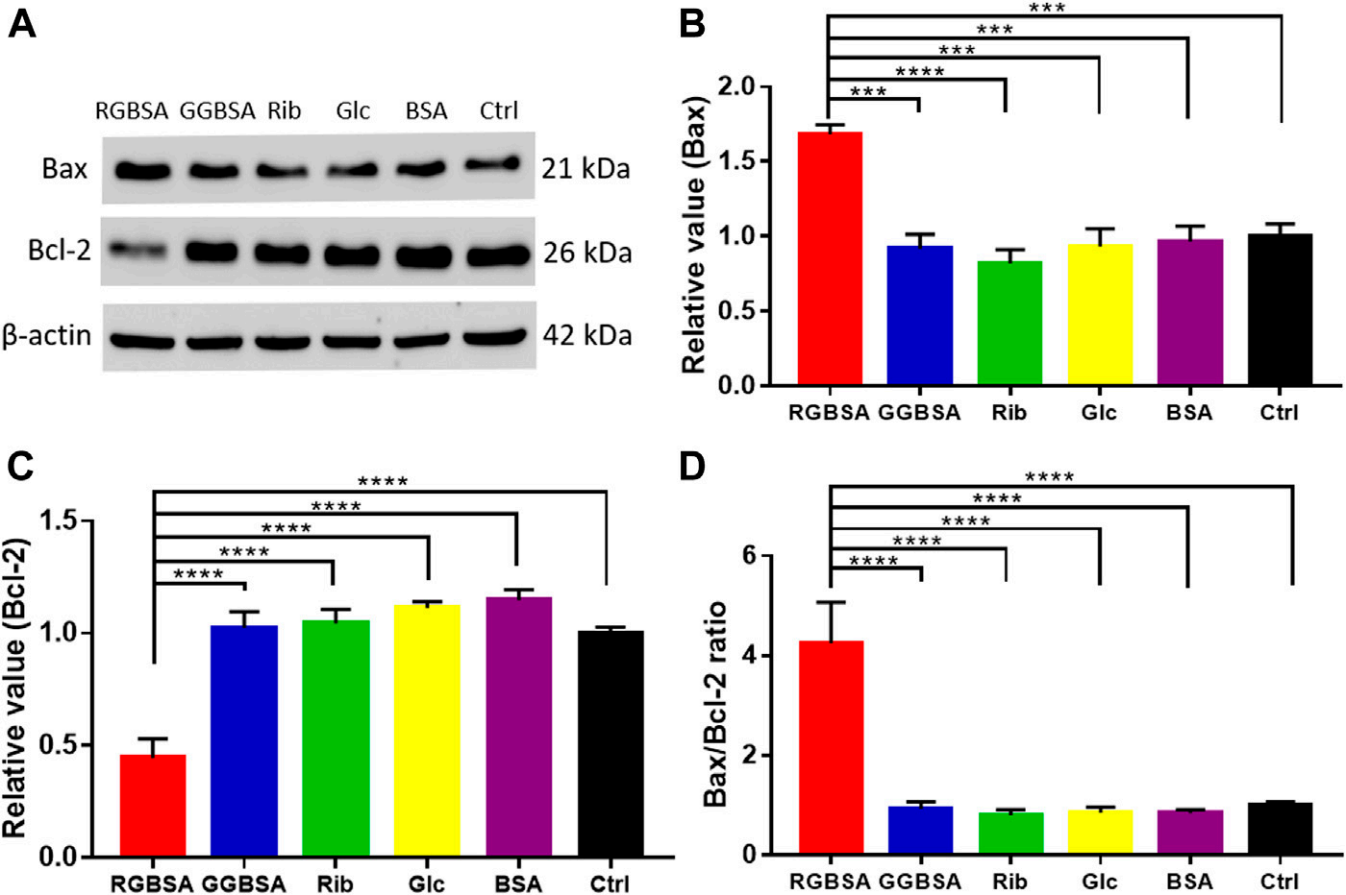
Riboglycated BSA regulates Bax and Bcl-2 proteins in SH-SY5Y cells. Experimental conditions were the same as in Figures 3C–H. The levels of Bax and Bcl-2 were determined by Western blot analysis **(A)**. β-actin was used as the loading control. Quantification results are shown in **(B,C)**. The control values were set at 1.0. The Bax/Bcl-2 ratios are shown in **(D)**. All data were analyzed by one-way ANOVA and are presented as the mean ± SEM (*n* = 4). ***p < 0.001 and ****p < 0.0001.

To further verify that riboglycated BSA induces apoptosis (Figure 3I), we evaluated the B-cell lymphoma-2 (Bcl-2) family of genes that regulate intrinsic apoptosis, consisting of both proapoptotic and antiapoptotic members, such as Bax and Bcl-2 (Banjara et al., 2020). The Bax/Bcl-2 ratio is used to determine the survival or death of cells (Korsmeyer et al., 1993). The Western blot analysis determined the expression levels of Bax and Bcl-2 in each group (Figure 4A). The proapoptotic Bax protein was significantly upregulated, but the antiapoptotic Bcl-2 protein was inhibited in SH-SY5Y cells after treatment with riboglycated BSA compared to the control cells (Figures 4B,C). Accordingly, the Bax/Bcl-2 ratio was significantly increased by approximately 4-fold after treatment with riboglycated BSA (Figure 4D). Therefore, these findings indicated that the riboglycated protein is more capable of inducing cell apoptosis than the glucoglycated protein. However, the mechanism of how riboglycated BSA causes cell death needs further investigation.

### Riboglycated bovine serum albumin increases lysine-specific demethylase-1 and plant homeodomain finger protein 8 leading to histone 3 demethylation on Lys 4

**FIGURE 5:**
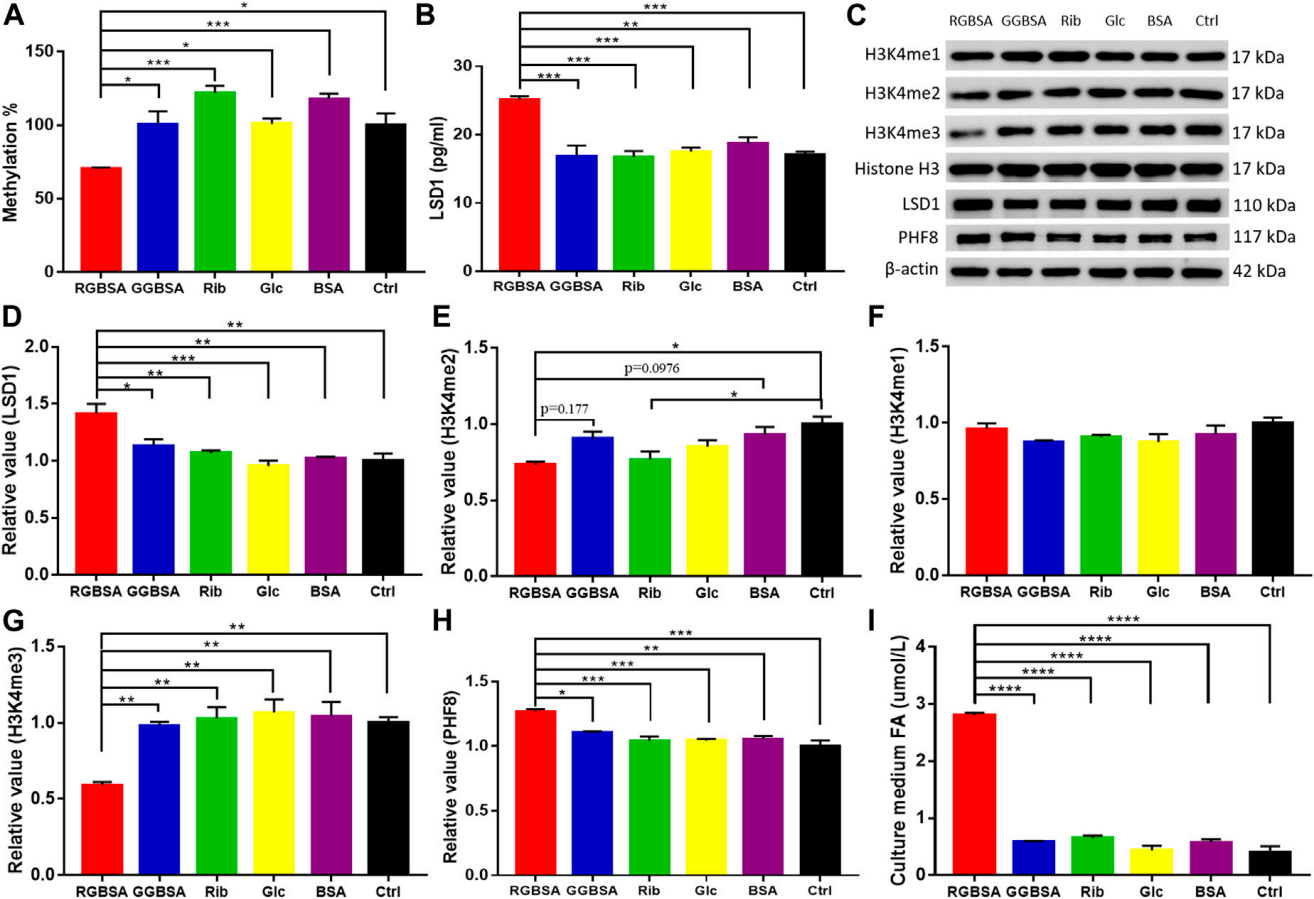
Riboglycated BSA reduces H3K4 methylation in SH-SY5Y cells. Experimental conditions were the same as in Figures 3C–H. The levels of global histone H3-K4 methylation **(A)** and LSD1 **(B)** were determined by ELISAs. The levels of H3K4me1, H3K4me2, H3K4me3, LSD1, and PHF8 in cells were determined by Western blot analysis **(C)**. Histone H3 or β-actin was used as the loading control. Quantification results are shown in **(D–H)**. The control values were set at 1.0. Quantitative assessment of culture medium FA levels detected by HPLC **(I)**. All values were analyzed by one-way ANOVA and are presented as the mean ± SEM (*n* = 3). *p < 0.05, **p < 0.01, ***p < 0.001, and ****p < 0.0001.

A delicate equilibrium in histone methylation and demethylation is required for proper gene regulation and maintenance of physiological metabolism (Jambhekar et al., 2019). Because changes in LSDs may lead to cellular metabolic disorders, we detected the levels of global histone methylation using ELISAs. A significant decrease in the H3-K4 methylation levels was observed in SH-SY5Y cells after incubation with riboglycated BSA compared to glucoglycated BSA (*p* ˂ 0.05) and the ribose (*p* ˂ 0.001), glucose (*p* ˂ 0.05), BSA (*p* ˂ 0.001), and Tris–HCl buffer (*p* ˂ 0.05) controls (Figure 5A). Because LSD1 plays an important role in histone de/methylation (Leiendecker et al., 2020), we measured LSD1 protein levels by ELISAs in SH-SY5Y cells. As shown in Figure 5B, the LSD1 levels were significantly increased in the presence of riboglycated BSA compared to glucoglycated BSA (*p* ˂ 0.001) and the controls (*p* ˂ 0.001 or *p* ˂ 0.01). The Western blot analysis (Figure 5C) demonstrated that LSD1 was significantly increased after treatment with riboglycated BSA but not after treatment with glucoglycated BSA or the controls (Figure 5D).

Because histone H3 peptide methylated at lysine 4 is a substrate for LSD1, we determined the methylation levels of H3K4me1, H3K4me2, and H3K4me3 in the SH-SY5Y cells in the presence of riboglycated proteins (Figure 5C). As shown in Figure 5E, there were increased H3K4me2 demethylation levels after treatment with riboglycated BSA compared to the Tris–HCl buffer control. However, the methylation levels of H3K4me1 did not significantly decrease for all the samples under the same conditions (Figure 5F). Interestingly, the demethylation of H3K4me3 was observed in the presence of riboglycated BSA but not glucoglycated BSA or the controls (Figure 5G). The marked demethylation of H3K4me3 may suggest that other enzymes in addition to LSD1 may be involved in the modification of H3K4me3 in the presence of riboglycated proteins. For example, PHF8 demethylates H3K9me1/2, and its catalytic activity is stimulated by adjacent H3K4me3 (Feng et al., 2010). Therefore, we measured PHF8 protein levels by Western blot analysis (Figure 5C) in SH-SY5Y cells. As shown in Figure 5H, the PHF8 levels were significantly increased in the presence of riboglycated BSA compared to glucoglycated BSA (*p* ˂ 0.05) and the controls (*p* ˂ 0.001 or *p* ˂ 0.01). These data suggested that LSD1 and PHF8 are both involved in H3K4 demethylation in the presence of riboglycated proteins.

### Treatment with riboglycated bovine serum albumin increases formaldehyde production of SH-SY5Y cells

Because formaldehyde is a consistent metabolic product of demethylation (Kalász, 2003), we detected the formaldehyde levels in the medium of SH-SY5Y cells cultured in the presence of riboglycated BSA, glucoglycated BSA, BSA alone, ribose alone, glucose alone, and Tris–HCl alone. As shown in Figure 5I, the formaldehyde levels were higher in the medium of cells cultured with riboglycated BSA (*p* ˂ 0.001) than the other treatments under the same experimental conditions. Thus, these data suggested that histone demethylation may act as an important source of cellular formaldehyde, which may affect the viability of SH-SY5Y cells.

## Discussion

According to many researchers (Chiou et al., 1999; Zhang et al., 2020; Wang et al., 2021), employing a high concentration of reducing sugar (1 M or higher glucose) in glycation studies is a routine method to accelerate the reaction *in vitro*. However, serum protein BSA after riboglycation, but not after glucoglycation, caused high cytotoxicity. The IC_50_, for the ability of riboglycated BSA to decrease SH-SY5Y cell viability, was 1.5 μM. Previous clinical studies have demonstrated that blood and urine ribose levels are significantly higher in both type 1 and type 2 diabetic patients than healthy individuals (Su et al., 2013; Chen et al., 2019; Yu et al., 2019; Mou et al., 2022). In addition, blood and urine ribose concentrations are positively correlated with HbA1c and glycated serum protein, respectively. In the present study, we observed a significant increase in LSD1 and PHF8 levels as well as in H3K4me2 and H3K4me3 demethylation levels in the presence of riboglycated BSA (1.5 μM final concentration) but not in the presence of glucoglycated BSA, suggesting that the riboglycated protein plays a role in the progression of diabetes with an epigenetic dysmetabolic disorder.

Hyperglycemia induces protein glycation, resulting in high levels of AGEs, which bind to RAGE and activate the RAGE pathway, ultimately inducing apoptosis *via* caspase 3 (Wu et al., 2018; Wu et al., 2019). The activation of the RAGE pathway leads to oxidative stress and evokes inflammatory reactions in numerous cell types and tissues, resulting in metabolic diseases (Guo et al., 2020). The present study demonstrated that riboglycation produced more AGEs at a faster rate than glucoglycation. As a monomer, we considered that riboglycated albumin binds to RAGE and triggers multiple cellular pathways, such as ROS elevation, Erk phosphorylation, NFκB activation, and BDNF/TiKB decline (Zhang et al., 2016; Dai et al., 2019; Wu et al., 2019). These cellular pathways are associated with caspase 3 activation and promote apoptosis, indicating that riboglycated BSA induces apoptosis through a RAGE-dependent pathway (Wei et al., 2016).

As mentioned previously, riboglycated BSA induced H3K4me2 and H3K4me3 demethylation but not H3K4me1 demethylation. The present study suggested that binding of PHF8 to H3K4me3 may stimulate the demethylation of H3K4me3 by KDM5A, resulting in decreased H3K4me3. H3K4me1 may be produced from demethylation of H3K4me2. However, LSD1 is a FAD-dependent histone demethylase with homology to amine oxidases, which demethylates di- and mono-methylated K4 on histone H3 (Hou and Yu., 2010; Kooistra and Helin 2012; Fiskus et al., 2014), thereby explaining H3K4me1 accumulation in the present study. In addition, LSD1 demethylation of H3K4me2 at glucocorticoid receptor (GR)-targeted enhancers is important for GC-mediated gene transcription, suggesting a molecular mechanism for H3K4me2 demethylation in gene activation (Clark et al., 2019). LSD1 binds to most GR-binding sites in the genome where it removes preprogrammed H3K4me2 but leaves H3K4me1 unchanged in cells. Thus, these results suggested that LSD1 and PHF8 are involved in the mechanism of riboglycated protein induction of oxidative stress.

Formaldehyde serves as a methyl donor in histone methylation (Li et al., 2021). Demethylation by both the KDM1 and JmjC demethylases oxidizes the methyl group, forming formaldehyde as a coproduct (Walport et al., 2012; Pavlenko et al., 2022). Therefore, we evaluated the formaldehyde concentration and found a significantly higher level of formaldehyde in the medium of SH-SY5Y cells cultured with riboglycated BSA than the other samples. In a complete catalytic cycle, the FAD cofactor is reduced to FADH2 and then is likely reoxidized by oxygen to produce hydrogen peroxide and formaldehyde. In general, formaldehyde is produced by more than one pathway in cells. For example, semicarbazide-sensitive amine oxidase (SSAO) catalyzes methylamine to produce formaldehyde (Yu et al., 2006), and mitochondria, as major cellular organelles, also produce formaldehyde (Xin et al., 2019). Considering that several pathways are involved in formaldehyde production, the present study suggested that H3K4 demethylation is one of the potential sources of formaldehyde.

Histone proteins are susceptible to glycation modifications due to long half-lives (for instance, 223 days in the brain) within cells and in the presence of abundant lysine and arginine residues (Commerford et al., 1982; Harmel and Fiedler., 2018; Rehman et al., 2022). Histone glycation occurs primarily on H3 and induces major chromatin changes *in vitro* and *in vivo*, and it has global ramifications on histone enzymatic PTMs and chromatin architecture as well as the assembly and stability of nucleosomes. The epigenetic impact of histone glycation was dependent on the sugar concentration and exposure time (Zheng et al., 2020a). Recent studies have suggested the interconnection of glycation with epigenetic PTMs, including histone methylation (Zheng et al., 2020b; Perrone et al., 2020), through competing sites, such as the substitution of glycation adducts for H3K4me3 and H3R8me2 (Zheng et al., 2019). As a non-enzymatic chemical modification, the occurrence of glycation is directly related to the concentration of the reactants and reaction time. However, demethylation occurs under enzyme-induced catalysis. It is well known that an enzymatic reaction is faster than a non-enzymatic reaction. Thus, further studies are required to determine whether ribose affects de/methylation due to the cross-talk between glycation and methylation of histones.

In conclusion, a low concentration (1.5 μM) of riboglycated BSA has higher cytotoxicity in SH-SY5Y cells than glucoglycated BSA. Treatment with riboglycated BSA induces demethylation of H3K4me3 and H3K4me2, which significantly increases the LSD1 and PHF8 levels, resulting in increased formaldehyde levels in cells. In addition, other histone demethylases, such as KDM5A, may also be involved in the mechanism of riboglycated protein-induced cell toxicity. The increased formaldehyde levels may also be attributed to other pathways in the presence of glycated BSA, thereby warranting further investigation.

## Data Availability

The original contributions presented in the study are included in the article/Supplementary Material. Further inquiries can be directed to the corresponding authors.
